# Transient and Prolonged Activation of Wnt Signaling Contribute Oppositely to the Pathogenesis of Asherman’s Syndrome

**DOI:** 10.3390/ijms23158808

**Published:** 2022-08-08

**Authors:** Xiang Xue, Xiaoli Li, Jinmeng Yao, Xue Zhang, Xu Ren, Shan Xu

**Affiliations:** 1 Department of Obstetrics and Gynecology, The Second Affiliated Hospital of Xi’an Jiaotong University, Xi’an 710004, China; 2 Department of Cardiology, The Second Affiliated Hospital of Xi’an Jiaotong University, Xi’an 710004, China; 3 Department of Obstetrics and Gynecology, The First Affiliated Hospital of Xi’an Jiaotong University, Xi’an 710004, China; 4 Core Research Laboratory, The Second Affiliated Hospital of Xi’an Jiaotong University, Xi’an 710004, China

**Keywords:** Asherman’s Syndrome, Wnt signaling pathway, endometrium, stem cells

## Abstract

Asherman’s Syndrome (AS) is caused by dysfunction of endometrial regenerative ability, which is controlled by adult stem cells and their niche. The Wnt signaling pathway has been demonstrated to be implicated in this process. This study aimed to clarify the relationship between the Wnt signaling pathway and the progression of AS after initial endometrial damage. Endometria with and without adhesion as well as from the intrauterine devices three months after the surgery were collected to compare the area of fibrosis. The area% of fibrosis did not vary significantly. Significantly higher expression of non-phosphorylated β-catenin, Wnt5a and Wnt7a was identified in the endometria with adhesion. The CD140b^+^CD146^+^ endometrial stem-like cells were present in the endometria with adhesion. Both Wnt5a and Wnt7a promoted stem cell proliferation. However, only Wnt7a preserved stem cell population by stimulating self-renewal. A rat endometrial injury model was established to investigate the effect of the activated Wnt/β-catenin signaling pathway on endometrial healing. We found that a transient activation of the Wnt/β-catenin signaling pathway promoted angiogenesis and increased the number of glands. In conclusion, transient activation of the Wnt/β-catenin signaling pathway during the acute endometrial damage may help the tissue regeneration, while prolonged activation may correlate to fibrosis formation.

## 1. Introduction

The endometrium is one of the most regenerative tissues in human body. Damage to the basal layer may cause failure in tissue regeneration and subsequently lead to Asherman’s Syndrome (AS) or intrauterine adhesion (IUA). In IUA, normal endometrium is replaced by fibrosis and connective tissue [1]. The distinct endometrial–myometrial junction is lost. In some extreme cases, only a single layer of epithelial cells is left so that the endometrium is no longer responsive to estrogen and progesterone [1]. After the current treatment, trans-cervical resection of adhesions (TCRA) followed by estrogen and progesterone cycle, the recurrence rate among the severe IUA cases is still high. Thus, recovering the endometrial regenerative ability is crucial to patients with severe IUA.

The remarkable regenerative ability of human endometrium is driven by adult stem cells. Both epithelial and stromal stem/progenitor cells have been identified in human endometrial tissue [2,3,4,5]. For both types, surface markers have been reported that enrich for stem cell activity. N-cadherin and SSEA-1 were identified as markers for stem cells from basalis epithelial cells [6,7]. SUSD2 [8], also known as W5C5, and double expression of CD140b and CD146 [3] were identified as mesenchymal stem-like cell markers. The selected cells generated more colony forming units (CFUs) and differentiated into multiple lineages. Previously, it was found that endometrial basalis is the stem cell pool [7,9,10]. Therefore, damage to endometrial basalis may lead to extensive loss of stem cells, which subsequently impairs the regenerative ability of the tissue. Previous findings suggest that the special menstruation niche could activate the stem cells as well as the canonical Wnt signaling pathway in the stem cells [11]. This indicates that the stem cell niche and canonical Wnt signaling pathway may play an important role in stem cell activation [12]. The components in the mimicked menstruation niche included several elevated CXC chemokines. The CXC chemokine expression in human mesenchymal stem cells (MSCs) is controlled by secreted frizzled-related proteins through the non-canonical Wnt signaling [13]. It is possible that the cyclic activation of the endometrial stem cells is also controlled in part by the non-canonical Wnt signaling.

The canonical Wnt signaling pathway has always been crucial to adult stem cell population expansion and self-renewal in several mammalian organs [14]. Both proliferative ability and self-renewal of MSCs are controlled by canonical Wnt signaling [14,15]. In neonatal mice, Wnt signaling-depending LGR5^+^ stem cells are essential to the development of endometrial glands [16]. In human endometrium, a variety of Wnt ligands are present, such as Wnt2, Wnt3, Wnt4, Wnt5a, Wnt7a and Wnt8b [17]. The Wnt signaling also participates in human endometrial growth and regression [18]. The canonical Wnt signaling activity changes cyclically across the menstrual cycle [19,20] and it can be activated in the endometrial mesenchymal stem-like cells by the mimicked menstruation niche [11]. This is also when the regeneration of the endometrium starts. Therefore, it is logical that the regeneration of the endometrium after damage is at least partially regulated by canonical/non-canonical Wnt signaling. We hypothesized that the canonical/non-canonical Wnt signaling in the endometrium with IUA is dysregulated. Altering the state of the canonical/non-canonical Wnt signaling pathway in the endometrial stem cells could result in self-renewal or differentiation. Activating the canonical Wnt signaling pathway at an early stage of endometrial injury contributes to the endometrial regeneration.

## 2. Results

### 2.1. Tissue Samples from IUA, Intra-Uterine Device (IUD) and Total Abdominal Hysterectomy (TAH) Displayed Different Histology Characteristics and Contained Similar Amount of Collagen

IUA samples were floating pieces of endometrial tissue during the TCRA surgery. IUD samples were tissues attached to the device. Both types of samples are from superficial endometrium and are extremely fragile. The majority of the samples were lost during the tissue processing procedure, which makes the two types of samples extremely precious.

**Figure 1:**
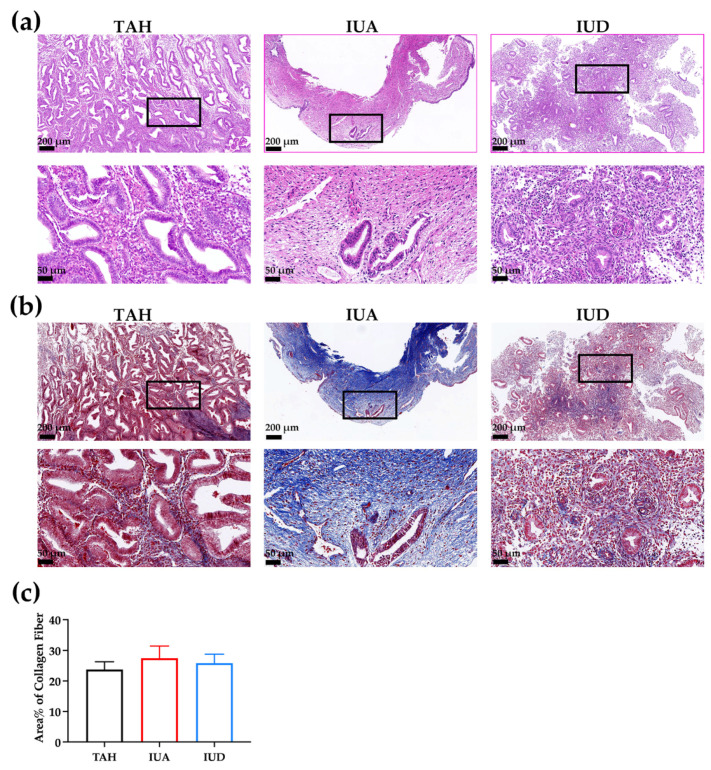
Sections of endometria from TAH, IUA and IUD of patients who received TCRA within the past three months were stained with H&E (**a**) and Masson trichrome staining (**b**). (**c**) Area% of collagen fiber based on the Masson trichrome staining is presented in bars. Magnified areas are marked in black boxes. Scale bar, 200 μm (top) and 50 μm (bottom/magnified). Data are expressed as mean ± SEM, (*n* = 5).

From both the H&E stain and Masson trichrome staining, we could see the IUA and IUD samples contain more red blood cells (Figure 1a) than the TAH endometrium. All the IUD samples we collected contained glands and stroma. The morphology of the glands, the number of blood vessels and the density of the stromal cells resemble that of the TAH endometrium. However, in a few IUA samples, glands were completely absent and significantly fewer blood vessels could be seen.

The fibrosis of the endometria of the patients before and after the surgery was evaluated (Figure 1b). Endometrial samples from TAH, IUA and IUD groups were stained with Masson trichrome staining. The fibrosis was interpreted by the percentage of the collagen fiber. However, the data did not reach statistical significance (Figure 1c) (TAH: 23.72% ± 2.55%; IUA: 27.42% ± 4.04%; IUD: 25.82% ± 2.92%; *n* = 5).

### 2.2. Canonical Wnt Signaling Activity Is Significantly Higher in the Endometria with IUA

**Figure 2:**
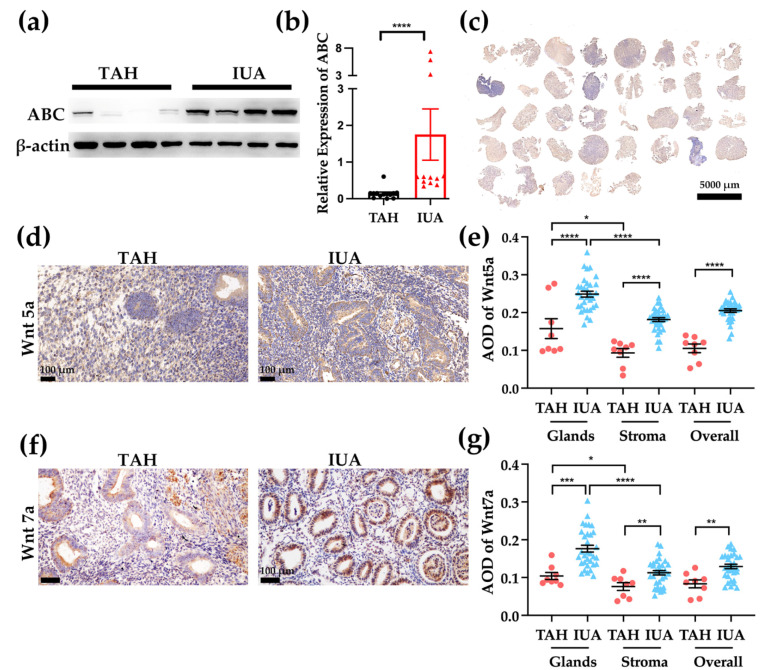
(**a**) The tissue array of endometria with IUA from 37 patients was established. Scale bar, 5 mm. (**b**) Western blot analysis was performed to examine the expression level of non-phosphorylated β-catenin (ABC, active β-catenin) in endometria of TAH and IUA patients. (**c**) Relative quantification of ABC normalized to β-actin (TAH *n* = 13; IUA *n* = 12). (**d**) Endometrial sections were stained with anti-Wnt5a using IHC. (**e**) Average optical density (AOD) of Wnt5a stained by IHC is presented in dots (TAH *n* = 8; IUA *n* = 32). Scale bar, 100 μm. (**f**) Endometrial sections were stained with anti-Wnt7a using IHC. Scale bar, 100 μm. (**g**) AOD of Wnt7a stained by IHC is presented in dots (TAH *n* = 8; IUA *n* = 32). Data are presented as mean ± SEM. * *p* < 0.05, ** *p* < 0.01, *** *p* < 0.001, **** *p* < 0.0001.

To evaluate the canonical Wnt signaling activity in the endometrium of AS patients, we performed a western blot assay to assess the expression of the non-phosphorylated β-catenin (ABC active β-catenin). The expression of the active β-catenin was significantly higher in endometria with IUA (Figure 2a,b, TAH: 0.087 ± 0.027, *n* = 13; IUA: 0.55 ± 0.083, *n* = 12, *p* < 0.0001).

### 2.3. Both Wnt5a and Wnt7a Expressions Are Higher in Endometrial Glands and IUA Endometrium

To investigate the expressions of Wnt5a and Wnt7a in normal endometrium and IUA endometrium, IHC was performed on tissue array of the endometrium with IUA (Figure 2c) and TAH sections. In full-thickness endometrium (TAH samples), Wnt5a and Wnt7a were present in both glands and stroma (Figure 2d,f). The expression of Wnt5a is higher in the glands (AOD 0.16 ± 0.03) than the stroma (AOD 0.093 ± 0.012) (Figure 2e), *n* = 8, *p* < 0.05. The endometria from IUA group showed a similar trend (the AOD of glands 0.25 ± 0.0079, the AOD of stroma 0.18 ± 0.0047, *n* = 32, *p* < 0.0001). When comparing the expression of Wnt5a in TAH and IUA endometrium, we found that both glandular expression and stromal expression of Wnt5a were significantly higher in the IUA endometrium (glands: *p* < 0.0001; stroma: *p* < 0.0001). Thus, the overall expression of Wnt5a was higher in the endometrium from the IUA patients (AOD of IUA: 0.20 ± 0.0047; AOD of TAH: 0.10 ± 0.017; *p* < 0.0001).

The expression pattern of Wnt7a is similar to Wnt5a. The glandular expression is significantly higher than the stroma for both TAH and IUA endometria (Figure 2g, TAH endometria: glandular AOD 0.10 ± 0.0094; stromal AOD 0.076 ± 0.010, *n* = 8, *p* < 0.05; IUA endometria: glandular AOD 0.18 ± 0.0090; stromal AOD 0.13 ± 0.0059, *n* = 32, *p* < 0.0001). The expression in the IUA endometria is higher than that in the TAH endometria (glands: *p* < 0.001; stroma: *p* < 0.01; overall: *p* < 0.01).

### 2.4. Both Wnt5a and Wnt7a Increased the Cloning Ability of the Endometrial Stem Cells and Wnt7a Could Maintain the Phenotype of This Population

**Figure 3:**
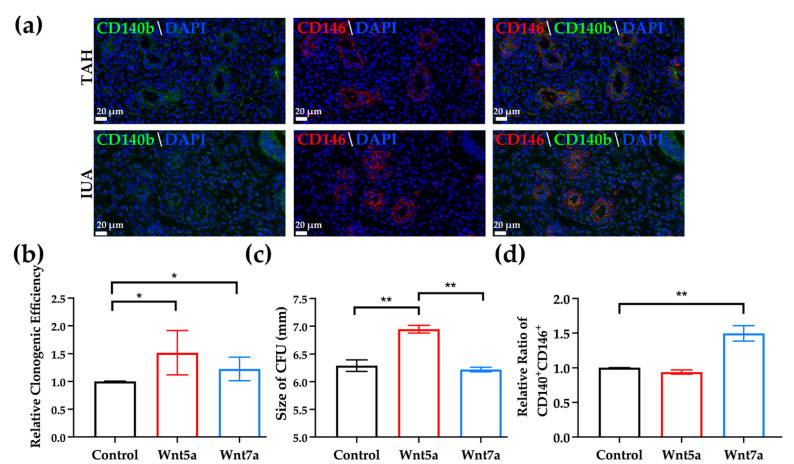
(**a**) Endometrial sections were stained with anti-CD140b (green), anti-CD146 (red) and DAPI (blue). CD140b and CD146 co-localization indicates population with enriched endometrial stem cells. The characteristics of CD140b^+^CD146^+^ population with supplemented recombinant Wnt5a or Wnt7a were examined. Scale bar, 20 μm. Relative cloning efficiency (**b**), size of the CFUs (**c**) and relative ratio of the population with co-expression of CD140b and CD146 (**d**) were evaluated. Data are presented as mean ± SEM (*n* = 5). * *p* < 0.05, ** *p* < 0.01.

Previously, it was demonstrated that the stem cell niche at the menstrual phase could promote the cloning ability and the self-renewal of the endometrial mesenchymal stem-like cells by activating the canonical Wnt signaling pathway [11]. We performed immumofluorescent staining and confirmed the presence of the CD140b^+^CD146^+^ stem cell population in the sample of IUA (Figure 3a).

In order to determine the impact of activating canonical and non-canonical Wnt signaling pathway on the endometrial stem cells (ESCs), we examined the effect of recombinant Wnt5a and Wnt7a on the stem cell culture. Wnt5a activates both canonical and non-canonical Wnt signaling while Wnt7a activates canonical Wnt signaling. Both Wnt5a and Wnt7a increased the relative cloning efficiency of the ESCs (Figure 3b, Wnt5a: 1.52 ± 0.18 fold, *n* = 5, *p* < 0.05; Wnt7a: 1.23 ± 0.095 fold; *n* = 5, *p* < 0.05). The diameters of the CFUs were also measured. The diameters of the CFUs in Wnt5a group were significantly larger than the control and Wnt7a group (Figure 3c, control 6.29 ± 0.10 mm; Wnt5a 6.95 ± 0.070 mm; Wnt7a 6.22 ± 0.041 mm; *n* = 5, *p* < 0.05). Only Wnt7a could maintain the phenotypic expression of the CD140b and CD146 double marker of the population (Figure 3d, Wnt5a 0.94 ± 0.03 fold; Wnt7a 1.50 ± 0.11 fold; *n* = 5, *n* = 5, *p* < 0.01).

### 2.5. Histological Evidence of Injury in the Rat Model of Endometrial Injury

**Figure 4:**
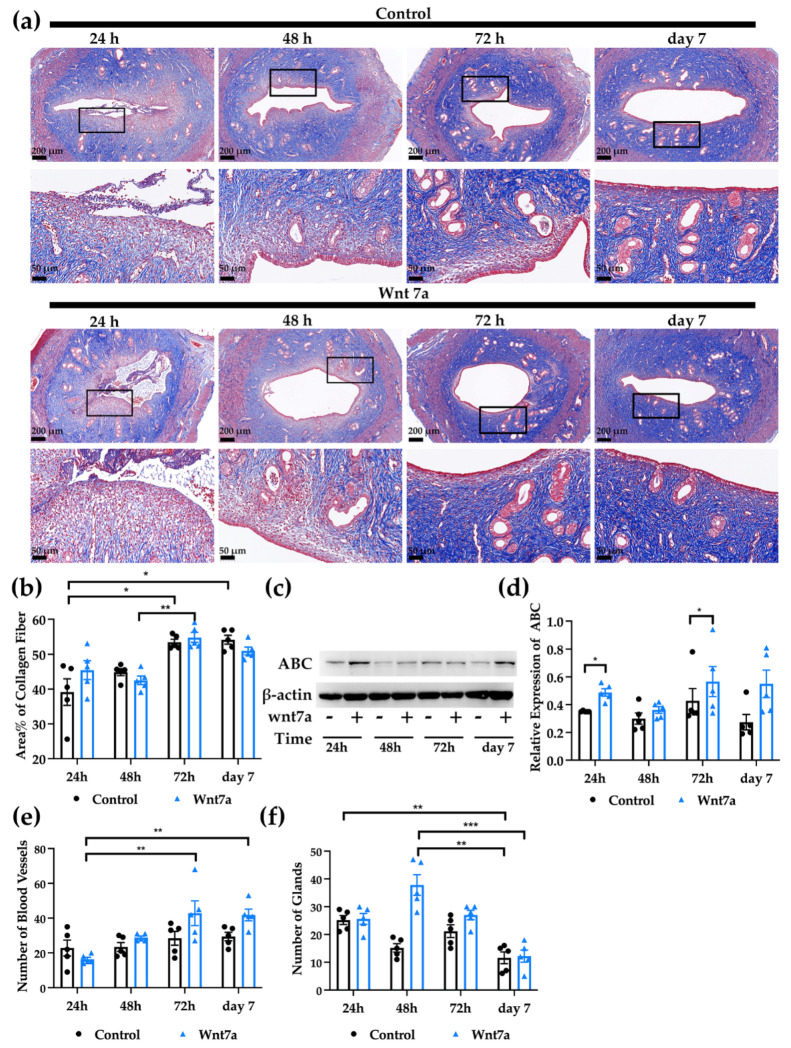
(**a**) Masson trichrome stained sections of rat uteri after induced injury with/without Wnt7a injection. Magnified areas are marked in black boxes. Scale bar, 200 μm (top) and 50 μm (bottom/magnified). (**b**) Area% of collagen fiber in the rat uteri is shown in bars. (**c**) Western blot analysis was performed to validate the activation of canonical Wnt signaling after injecting recombinant Wnt7a protein. (**d**) Relative quantification of ABC normalized to β-actin. Number of blood vessels (**e**) and glands (**f**) in the uteri per section at different times after the induced injury. Data are presented as mean ± SEM (*n* = 5). * *p* < 0.05, ** *p* < 0.01, *** *p* < 0.001.

To evaluate the rat model for endometrial injury, the uterine horns were collected at the corresponding time and stained with H&E and Masson trichrome staining. It was obvious that both luminal epithelium and the stroma were damaged during the surgery (Figure S1 and Figure 4a). At 24 h, the luminal epithelium was completely disrupted. The uterine lumen was filled with shredded tissue. Glands were ruptured. In the stroma, we could see stenosis of the lumen, tissue edema, necrosis, red blood cells and a significant amount of leukocytes. The luminal epithelium was restored completely within 72 h after the surgery.

The Masson trichrome staining showed that in control side uteri, the area% of fibrosis (collagen fiber) at 72 h and day 7 was significantly higher than 24 h (Figure 4a,b; 24 h: 39.13% ± 3.82%; 48 h: 44.88% ± 1.00%; 72 h: 53.47% ± 0.86%, *p* < 0.05; day 7: 54.16% ± 1.24%, *p* < 0.05). On the other hand, the uteri treated with recombinant Wnt7a protein were found with the highest area% of collagen fiber at 72 h, which was significantly higher compared to 48 h (24 h: 45.45% ± 2.69%; 48 h: 42.51% ± 1.24%; 72 h: 54.82% ± 1.39%, *p* < 0.01; day 7: 50.98% ± 1.11%).

Western blot analysis was performed to validate the activation of canonical Wnt signaling by the injection of Wnt7a (Figure 4c,d). The expression of non-phosphorylated β-catenin in the uteri with Wnt7a was higher than corresponding control side (24 h: control 0.35 ± 0.0020, Wnt7a 0.49 ± 0.026, *p* < 0.05; 48 h: control 0.30 ± 0.040, Wnt7a 0.36 ± 0.024; 72 h: control 0.43 ± 0.088, Wnt7a 0.57 ± 0.11, *p* < 0.05; day7: control 0.27 ± 0.055, Wnt7a 0.55 ± 0.099).

### 2.6. Activation of Canonical Wnt Signaling Pathway Supports the Growth of Blood Vessels and Glands after Endometrial Injury

Both blood vessels and glands were counted after the surgery of the rat uteri. The number of the blood vessels in the control uteri was similar at 24 h (22.80 ± 4.55), 48 h (23.40 ± 2.54), 72 h (28.40 ± 3.94) and day 7 (29.40 ± 2.44) (Figure 4e). For the uterine horns with recombinant Wnt7a protein, the blood vessels increased significantly from 24 h to day 7 (24 h: 16.40± 1.12; 48 h: 29.40 ± 0.98; 72 h: 42.80 ± 7.10; day 7: 41.80 ± 3.39; *p* < 0.01).

The number of glands varied significantly in both control and Wnt7a treated uteri. On control side, the glands declined significantly between 24 h and day 7 (Figure 4f, 24 h: 25.20 ± 1.59; 48 h: 15.20 ± 1.50; 72 h: 21.20 ± 2.29; day 7: 11.60 ± 2.11). For the uteri injected with recombinant Wnt7a protein, the gland number was highest at 48 h and decreased dramatically afterwards (24 h: 25.60 ± 1.96; 48 h: 37.80 ± 3.73; 72 h: 27.00 ± 1.67; day 7: 12.20 ± 2.18; *p* < 0.001). Moreover, the number of glands at 48 h in the uteri with Wnt7a was higher than in the control side at 48 h (*p* = 0.06) and day 7 (*p* < 0.01).

### 2.7. Wnt7a Recombinant Protein Reduced the Apoptosis of the Endometrial Cells Rapidly after the Uterine Damage

**Figure 5:**
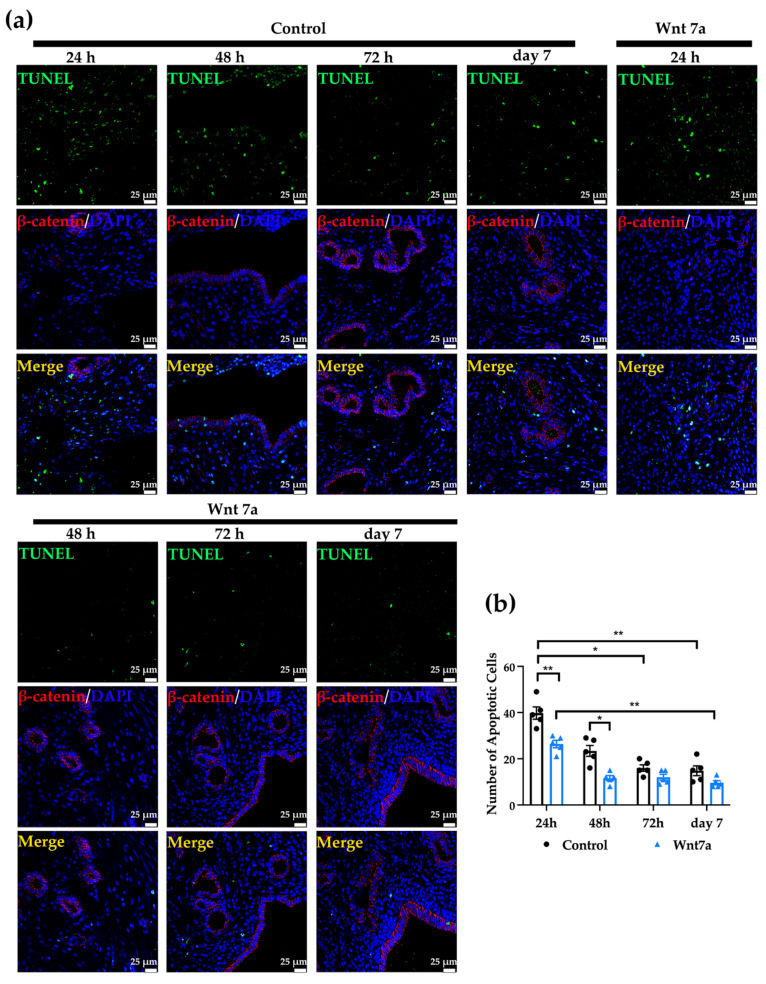
(**a**) The rat uteri were stained with DAPI (blue), TUNEL (green) and anti-non-phosphorylated β-catenin (red) to investigate the number of apoptotic cells and activation of canonical Wnt signaling at 24, 48, 72 h and 7 days after induced injury. Scale bar, 25 μm. (**b**) The number of apoptotic cells per section at different time after the induced injury is presented in bars. Data are presented as mean ± SEM (*n* = 5). * *p* < 0.05, ** *p* < 0.01.

TUNEL immunofluorescent staining was performed to evaluate the apoptotic cells after the induced damage of the endometrium in the rat model (Figure 5a). The overall number of apoptotic cells decreased over time after the injury. The number of apoptotic cells of control group at 24 h was significantly higher than 72 h and day 7 (Figure 5b. 24 h: 39.80 ± 2.65; 48 h: 23.40 ± 2.38; 72 h: 16.00 ± 1.38, *p* < 0.01; day 7: 14.80 ± 2.13, *p* < 0.01). The uteri with supplemented Wnt7a protein showed a similar trend as the control side. Specifically, the number of apoptotic cells at 24 h was significantly higher than day 7 (24 h: 26.40 ± 1.60; 48 h: 11.60 ± 1.12, *p* < 0.01; 72 h: 12.00 ± 1.26; day 7: 9.60 ± 0.93, *p* < 0.001). Comparing control side and Wnt7a treated side, we found that at both 24 and 48 h, the number of apoptotic cells was significantly higher in the control side (24 h, *p* < 0.01; 48 h, *p* < 0.05).

## 3. Discussion

The regenerative ability of the endometrium of Asherman’s Syndrome patients is only limited. Surgery aims at separating the adhesions in the uterine cavity, but does little to endometrial regeneration and much less on recurrence [21]. Therefore, for the severe AS cases, rebooting the endometrial regeneration is crucial. Therefore, we examined the effect of both Wnt5a and Wnt7a on the stem cells enriched by CD140b^+^CD146^+^.

We collected three types of samples, namely TAH, IUA and IUD, representing normal (adhesion-absent), adhesion and post-operative endometrium. This is the first study to compare the endometrium with IUA and from IUD after TCRA. Acquiring a proper tissue sample from the IUDs was extremely difficult. Approximately 80% of the patients either result in a clean IUD or a tissue which is too small to retrieve or survive the tissue processing. The Masson trichrome staining shows the area of collagen fibers, which partially indicates fibrotic tissue. Compared with endometria from TAH which contain the intact myometrium and epithelium, both IUA and IUD samples are loose since they are extremely superficial. This may lead to the insignificance in the area% of the collagen fiber by Masson staining. The fibrotic tissue of IUA patients could be deeper and thus impossible to acquire a sample from the patients.

Previously, it was reported that the expression of endometrial stem cell markers, CD140b and CD146, was significantly higher in the endometrium with IUA [22]. However, the stem cell population can only be enriched by double expression of the two markers. Thus, we checked the double expression of CD140b and CD146 in the endometrium with IUA. The majority of this population could be located in the perivascular location or close to glands. Since the canonical Wnt signaling is related to the activation of the endometrial stem cells [11,23,24], we also examined its activity in the endometrium of IUA patients. We found that non-phosphorylated β-catenin was significantly higher in the endometrium with IUA, indicating an abnormal and prolonged activation of the canonical Wnt signaling. Previously, Wnt signaling has been reported to contribute to stem cell aging and increase tissue fibrosis [25]. Other reports have also indicated the role of Wnt signaling in the ECM formation in the endometrium [26], which may also contribute to fibrosis formation. Thus, it is possible that the pathogenesis of IUA is partially due to hyper-activation of Wnt signaling, which leads to increased growth of the fibrotic tissue.

Subsequently, we examined the expression of Wnt5a and Wnt7a in the endometria with IUA. Wnt5a may activate both canonical and non-canonical Wnt signaling [27], while Wnt7a mainly activates canonical Wnt signaling [18,28]. Wnt7a has been shown to be expressed exclusively in the epithelial cells of the female reproductive tract [29,30]. Our results confirmed that both molecules are widely expressed by endometrial cells in IUA cases with the highest expression level in the glandular epithelial cells. This may cause the hyperactivation of the canonical Wnt signaling.

Our cell experiment shows that recombinant Wnt5a and Wnt7a both promoted the proliferation of the CD140b^+^CD146^+^ population. However, Wnt5a could enlarge the size of the CFUs without preserving the expression of the double marker. This indicates that as the stem cells proliferate, more cells lose their phenotype upon the stimulation of Wnt5a. This finding supports our hypothesis that hyper-activation of Wnt signaling contributes to the pathogenesis of IUA. Thus, it is interesting to investigate whether Wnt5a may lead to differentiation towards a specific lineage. Besides, the link between Wnt activation and endometrial fibrosis also remains to be confirmed.

It is worth mentioning that the age of IUA and TAH patients are significantly different. The IUA patients are younger and have needs of fertility. However, the patients undergoing TAH are generally closer to menopause. In our study, all the stem cells were isolated from TAH samples, since the IUA tissue fragments were too small for stem cell isolation. Thus, the demographic difference should not affect the results from the stem cell experiments. However, it has been reported that the activity of Wnt signaling is affected by aging. Wnt signaling activity is significantly down-regulated in aged bones [31], while up-regulated in aged kidney [32]. Although, in some organs, Wnt signaling changes with aging, and the influence of pathological conditions is usually more prominent [33]. Presently, a large number of research papers can be found about Wnt signaling and uterine pathologies. However, the relationship of endometrial Wnt signaling and aging is unclear. It would be an interesting topic to investigate. Understanding the role of Wnt signaling in the interaction between endometrium and aging may shed light on specific uterine pathogenesis.

Murine models of IUA have been well-established for many years and widely used in the study of Asherman’s syndrome. In this study, we combined both mechanical and chemical injury to create a model which resembles the severe damage to the endometrium. The effect of activating canonical Wnt signaling during the window of acute damage and the healing process is more of our interest. Besides, the intact luminal epithelium may be a barrier to compromise the effect of Wnt ligands. Thus, Wnt7a was injected immediately after the induction of the injury. The Masson trichrome staining suggests that for both groups, fibrosis forms at 72 h after the injury. The number of blood vessels increased gradually over 7 days. However, the increase on control uteri was not as significant as the Wnt7a injected side. This suggests that activation of canonical Wnt signaling contributes to the endometrial angiogenesis. Both canonical and non-canonical Wnt signaling are implicated in vascularization in a variety of organs such as the central nervous system, eyes and tumors [34,35,36]. It is not surprising that Wnt signaling is involved in the endometrial angiogenesis after injuries.

Without Wnt7a, the number of glands at day 7 was significantly lower than 24 h. A similar trend was noticed for the Wnt7a injected side. This is consistent with the trend of the formation of the fibrosis, when significant amount of collagen fibers starts to form after 72 h. However, Wnt7a significantly increased the number of glands at 48 h. This suggests that activation of Wnt signaling helped endometrial restoration within a relatively short period after the injury. The follow-up formation of the fibrosis and reduction of the glands may be due to the exhaustion of Wnt7a or hyper-activation of the fibrinogen aggregation. This remains to be investigated. We also found that the intrauterine injection of Wnt7a significantly reduced the apoptosis of the cells, especially at 24 and 48 h. More interestingly, we seldom saw any co-localization of TUNEL and ABC in the sections during the quantification. This further supports that activation of Wnt signaling is implicated in tissue repair at the early stage after the endometrial damage. Unfortunately, without surface markers, it is almost impossible to isolate stem cells from rat uteri. Therefore, it is difficult to verify the effect of Wnt7a on endometrial stem cells in the murine models.

Our findings indicate that there may be a contradiction in the effect of Wnt signaling pathway regarding the healing after endometrial injury. Initial healing and formation of the fibrosis are two separate physiological processes. The intervention time of Wnt signaling after the damage may be important. The difference of Wnt5a and Wnt7a in stem cell differentiation and self-renewal suggests that the canonical and non-canonical Wnt signaling pathway may play different roles in endometrial stem cell regulation.

## 4. Materials and Methods

### 4.1. Human Tissues

Three types of tissue samples were collected, namely, full thickness endometria from total abdominal hysterectomy (TAH), IUA endometria and the endometria from the intra-uterine device (IUD) of the patients who had received TCRA within the past three months. All participants of TAH and IUA group had not taken hormonal therapy for at least 3 months prior to the surgery and they had been excluded for polycystic ovary syndrome. Full thickness endometrial samples (TAH samples) were acquired from women who underwent total abdominal hysterectomy for benign pathologies, such as endometriosis and myoma. A total number of 14 women with regular menstrual cycles were recruited (median age 45; range 42 to 51 years). A total number of 44 women with IUA were recruited (median age 31; range 25 to 39 years). To acquire IUA samples, no scrape or resection was done purposely. The IUA samples were acquired by holding a gauze at the outflow of the distention media during surgery. Tissue samples were collected from the gauze. Five IUD samples were collected (median age 30; range 26 to 32 years). These tissues were acquired from the IUD when patients revisited the clinic to remove the device three months after TCRA. All the samples were categorized into two phases, namely, proliferative (*n* = 29) and secretory (*n* = 34), based on the date of the collection at the menstrual cycle or hematoxylin-eosin stain.

### 4.2. Single-Cell Isolation of Endometrial Cells

Endometrial single-cell isolation was carried out as described [9]. Endometrial tissues were collected and the following procedure was performed with 24 h of the collection. Tissues were minced into pieces smaller than 1 mm and suspended in PBS containing collagenase III (0.3 mg/mL, Worthington, Lakewood, NJ, USA) and deoxyribonuclease I (40 ug/mL, SolarBio, Beijing, China). Tissue suspension was digested at 37 °C in water bath under constant shaking. Red blood cells were removed by density-gradient centrifugation using Ficoll-Paque (GE Healthcare, Berlin, Germany). Leukocytes were excluded using anti-CD45 microbeads (Miltenyi Biotec Inc., Bergisch Gladbach, Germany). Negative cells were subjected to subsequent isolation for epithelial and stromal cells. Epithelial cells were selected using anti-CD326 microbeads (Miltenyi). Negative cells were considered as stromal cells. Stromal cells were cultured in fibronectin-coated plates (1 mg/mL, Invitrogen). Culture media contained DMEM/F12, 10% FBS, 1% antibiotics, and 2 mmol/L L-glutamine (Invitrogen, Carlsbad, CA, USA). Cell culture was incubated in a humidified carbon dioxide (5%) incubator at 37 °C.

### 4.3. Isolation of Endometrial Stem-like Cells

Endometrial stem cells were isolated by previously described magnetic beading method [9]. In short, stromal cells were first incubated with PE-conjugated anti-CD140b (R&D System, Minneapolis, MN, USA) antibody followed by anti-mouse IgG1 microbeads (Miltenyi Biotec Inc.). After incubation, cells were applied to Miltenyi MS columns with magnetic fields to isolate CD140b^+^ population. Afterwards, cells were expanded for 7 days followed by another round of beading with CD146 microbeads (Miltenyi Biotec Inc.). CD140b^+^CD146^+^ population (endometrial stem-like cells, ESCs) were subjected to following experiments.

### 4.4. Addition of Recombinant Proteins

ESCs were seeded at clonegenic density in 6-well plates (300 cells per well). Recombinant human Wnt5a (0.1 μg/mL, R&D system, Minneapolis, MN, USA) and Wnt7a (0.1 μg/mL, PeproTech, Cranbury, NJ, USA) were added to the culture media of ESCs. Fresh culture media with corresponding recombinant proteins were added on day 7. Colony forming ability and co-expression of the ESC markers were recorded on day 14.

### 4.5. Colony Forming Ability

The ESCs were seeded at clonegenic density in 6-well plates (300 cells per well). Fresh media was supplemented at day 7. The number of CFUs were recorded on day 14. The size of the CFU was defined by its diameter. Those with a diameter over 6 mm are defined as large.

### 4.6. Co-Expression of ESC Markers (CD140b and CD146)

Flow cytometry analysis was performed to evaluate the co-expression of ESC markers. Endometrial cells were labelled with phycoerythrin (PE)-conjugated anti-CD140b antibody (R&D System) and fluorescein isothiocyanate (FITC)-conjugated anti-CD146 antibody (Thermo Fisher Scientific, Waltham, MA, USA), or isotype controls. Cells were incubated with antibody for 45 min at 4 °C in 0.1% BSA/PBS for labeling. After incubation, cells were washed and preserved in 0.1% BSA/PBS until analysis. Flow cytometry analysis was performed using the FACS Aria II (BD Biosciences, Bedford, MA, USA). Results were analyzed using FlowJo software 10.8.1 (Tree Star, Ashland, OR, USA).

### 4.7. Tissue Processing

Tissues were fixed by 4% PFA upon collection for 4–12 h depending on the size of the tissue. After fixation, tissues were preserved in 80% ethanol. Upon processing, they were dehydrated in increasing concentration of ethanol (95% ×2, ethanol ×2). After dehydration, they were cleared in xylene and paraffinized at 65 °C in three melted paraffin jars, namely, 1 h, 40 min, 30 min. Afterwards, the tissues were embedded using a Leica EG 1150 machine.

### 4.8. Tissue Array Construction

Paraffin blocks of 37 IUA cases were selected. For each case, 4 μm sections were stained with H&E to select designated areas for tissue array. After review, an area was encircled on the slide. A core of 5 mm diameter was taken from the marked area and placed in a recipient blank paraffin block on pre-designated array location. The finalized array block was cut into 4 μm sections and subject to further stains.

### 4.9. Hematoxylin-Eosin Stain

The tissues were cut into 4 μm sections and deparaffinized in xylene and rehydrated in decreasing concentration of ethanol baths. After hydration, the nuclear was stained in hematoxylin for 5 min and washed in running tap water for 3 min. Afterwards, the sections were differentiated in 1% HCl/EtOH for 30 s and rinsed in tap water. Subsequently, the slides were dipped in ammonia water for 2 min until sections become blue, followed by tap water rinse. Finally, they were counterstained in Eosin Y for 20 s. After staining, the tissue sections were dehydrated in increasing concentration of ethanol and cleared in two xylene baths before mounting. The slides were scanned using the Pannoramic MIDI digital scanner (3DHISTECH Ltd., Budapest, Hungary) and then viewed using the CaseViewer 2.4 (ServiceBio, Wuhan, China).

### 4.10. Masson Trichrome Staining

The paraffin blocks were cut into 4 μm sections and deparaffinized in xylene and rehydrated in decreasing concentration of ethanol baths. The Masson trichrome staining kit (SolarBio) was used according to manufacturer’s instruction for the staining. After rehydration, sections were stained with Weigert’s iron hematoxylin solution for 10 min followed by tap water rinse. Afterwards, the slides were stained with Beibrich-Scarlet Acid Fuschin solution for 15 min and washed in distilled water. Then, the color was differentiated in phosphomolybdic-phosphotungstic acid solution for 10 min and the sections were subsequently transferred to aniline blue solution for 5 min. Finally, the slides were rinsed in distilled water briefly and differentiated with 1% acetic acid solution for 3 min followed by a rinse by distilled water. After the trichrome stain, the sections were dehydrated, cleared and mounted for observation. All slides were scanned using the Pannoramic MIDI digital scanner (3DHISTECH Ltd., Budapest, Hungary) and then viewed using the CaseViewer 2.4 (ServiceBio, Wuhan, China). Results were analyzed by ImageJ software and the percentage area (%Area) was used to interpret the amount of the collagen fibers in the slides.

### 4.11. Immunohistochemistry (IHC)/Immunofluorescence (IF)

Upon staining, the sections were deparaffinized in xylene and rehydrated in decreasing concentration of ethanol. Heat-induced antigen retrieval was performed in sodium citrate-EDTA antigen retrieval solution (Beyotime Biotechnology, Shanghai, China) in a microwave oven. For IHC staining, endogenous peroxidase activity was blocked with 3% H_2_O_2_ for 10 min. Primary antibody anti-Wnt5a (ABclonal, Wuhan, China) or anti-Wnt7a (Proteintech Group, Wuhan, China) was incubated at 4 °C overnight and secondary antibody was incubated after washing in PBS on the following day. IHC sections were subsequently stained with hematoxylin before dehydration and mounting while IF sections were stained with 4′,6-diamidino-2-phenylindole (DAPI, Thermo Scientific, Waltham, MA, USA) before mounting. Slides were scanned using the Pannoramic MIDI digital scanner (3DHISTECH Ltd., Budapest, Hungary) and then viewed using the CaseViewer 2.4 (ServiceBio, Wuhan, China). The IHC stained slides were evaluated by Image Pro Plus (Media Cybernetics, Inc., Bethesda, MD, USA) to acquire the integrated optical density (IOD). Average optical density (AOD) was calculated by IOD/area. The AOD of glands and stroma were measured separately. An overall AOD of the picture was also acquired.

### 4.12. Terminal Deoxynucleotidyl Transferase Biotin-dUTP Nick End Labeling (TUNEL) Assay

Apoptosis in rat endometrium was detected by TUNEL staining. To prepare paraffin sections, the rat uteri were collected and fixed by 4% PFA upon collection. All the tissues were processed as described above. The 4 μm tissue sections were cut using a rotary microtome (Leica RM2235, Nussioch, Germany). Sections were deparaffinized, rehydrated, treated with protease and permeabilized. Subsequently, they were blocked with 5% BSA for 30 min and washed by PBS. Primary antibody non-phosphorylated β-catenin (Cell Signaling Technology, Boston, MA, USA) was incubated overnight at 4 °C. The slides were washed with PBS the next day followed by secondary antibody staining (goat anti-rabbit AlexaFluor 555, Thermo Fisher Scientific, Waltham, MA, USA). Subsequently, the sections were labelled using the in situ cell death detection kit according to manufacturer’s instruction (Roche, Penzberg, Germany). In short, TUNEL reaction mixtures were prepared accreting to the protocol. The slides were rinsed and incubated with reaction mixture or control reagent, and incubated at 37 °C for 60 min. After incubation, they were washed in PBS to remove excessive reaction reagent. The sections were subsequently stained with DAPI for 5 min to visualize the nuclei. All slides were then mounted in antifade mounting media and subject to visualization under confocal microscopy (Leica TCS SP8 CARS, Wetzlar, Germany).

### 4.13. Animal Uterine Damage Model

Twenty SD rats, aging 6–8 weeks, weighing 200–250 g was randomly selected and housed in the laboratory animal center of Xi’an Jiaotong University. Animals were housed in the facility under standard environmental conditions (25 ± 1 °C, 55  ±  5% humidity and 12 h/12 h light/dark cycle) and had access to food and water ad libitum. All rats were subjected to pre-operative overnight fasting. Before surgery they were anesthetized with isoflurane (3%, oxygen flow 3 L O_2_/min). Animals were shaved and sterilized before a 2 cm incision was made at 2–3 cm above the symphysis pubis. The Y-shape uteri were then exposed. To induce damage, the uterine horn was picked up by forceps and an 18 G needle was inserted from the distal end until reaching the cervix. The needle was then rotated and pulled out to its entrance. The scraping procedure was repeated three times. No damage was made to the serosa. After the scraping, 10 μL ethanol was injected into the same side to further induce the damage. Ethanol was kept in the uterine cavity for 10 s before washed with saline. After inducing the injury, the right uterine horns were given 5 μL Wnt7a recombinant protein (0.5 μg/mL). They were put back and the peritoneal cavity was rinsed with saline before closure. At post-operative 24, 48, 72 h, and 7 days, rats were sacrificed on each time point by overdosing with 2% pentobarbital sodium followed by dislocation of the neck. Uterine horns were collected and fixed in 4% PFA or snap frozen in liquid nitrogen for protein analysis. To evaluate the number of blood vessels and glands in the uteri, three different sections (at least twenty sections away from each other) were stained with H&E or Masson trichrome staining. The average number of blood vessels or glands was calculated.

### 4.14. Western Blot

Proteins were released from the tissue using RIPA. 1% phenylmethanesulfonyl fluoride (PMSF, Roche, Mannheim, Germany) and proteinase inhibitor (Roche, Germany) was added into each reaction. Tissue debris was removed by centrifugation at 14,000 rpm. Protein concentration was determined using bicinchoninic acid (BCA) protein assay (Beyotime, Shanghai, China) following the manufacturer’s instruction (KeyGen Biotech Co., Ltd., Nanjing, China). The extracted proteins were mixed with 5× SDS loading buffer (60 mM Tris-HCl, pH 6.8, 2% SDS, 0.1% bromophenol blue, 25% glycerol and 14.4 mM β-mercaptoethanol) and denatured at 95 °C for 10 min. The protein samples (40 μg/sample) were electrophoresis on 10% SDS-polyacrylamide gels and transferred to polyvinylidene difluoride (PVDF) membranes (Millipore, Billerica, MA, USA). The membrane was blocked with 5% skim milk with 0.1% tween-20 in PBS for 1 h at room temperature and incubated with primary antibodies against non-phosphorylated β-catenin (Cell Signaling Technology, Danvers, MA, USA) at 4 °C overnight, or β-actin (Proteintech Group, Wuhan, China) at room temperature for an hour. Secondary antibodies were incubated on next day for 1 h at room temperature. WesternBright kit (Millipore Corporation, Billerica, MA, USA) was used to visualize the protein bands. The optical density of the bands was measured by ImageJ software, and the values were normalized to β-actin.

### 4.15. Statistical Analysis

Data were analyzed using GraphPad PRISM software 9 (GraphPad Software, San Diego, CA, USA). All continuous variables were tested for normality using D’Agostino and Pearson test. Paired or unpaired *t*-test was used to determine the statistical significance between two groups if the data were normally distributed. If not, Mann–Whitney or Wilcoxon test was used for unpaired or paired data respectively. For results with more than two groups, Kruskal–Wallis test followed by Dunn’s post-test was used if the data did not pass normality test. All data are presented as mean ± SEM. *p* < 0.05 was considered statistically significant.

## 5. Conclusions

Our findings indicate that the prolonged activation of the canonical Wnt signaling pathway in the endometria may correlate to the pathogenesis of IUA. Both Wnt5a and Wnt7a expression were higher in the endometria with IUA. However, they were implicated differently in the endometrial stem cell self-renewal and differentiation. The canonical Wnt signaling promoted the proliferation and self-renewal of the stem cells, while the non-canonical Wnt signaling only stimulated proliferation. Activation of Wnt/β-catenin signaling pathway immediately after uterine injury contributes to endometrial regeneration. However, prolonged activation of Wnt signaling may facilitate the formation of fibrosis.

## Supplementary Materials

The following supporting information can be downloaded at: https://www.mdpi.com/article/10.3390/ijms23158808/s1.
