# Supplementary figures and images for: Transient and Prolonged Activation of Wnt Signaling Contribute Oppositely to the Pathogenesis of Asherman’s Syndrome
Source: Int J Mol Sci. 2022 Aug 8;23(15):8808. doi: 10.3390/ijms23158808 (PMC9368949; doi:10.3390/ijms23158808)

## Control

24 h

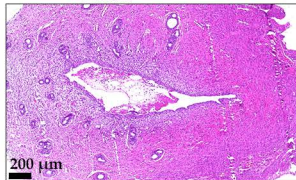

48 h

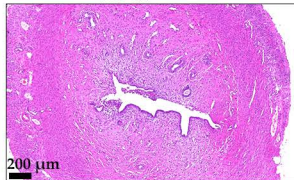

72 h

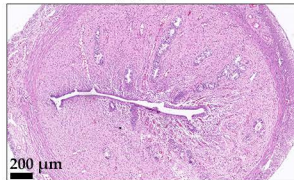

day 7

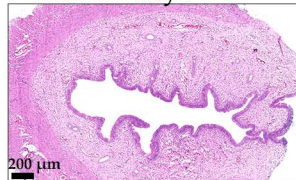

## Wnt 7a

24 h

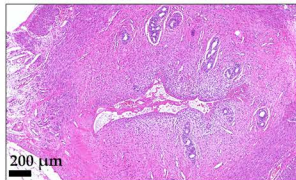

48 h

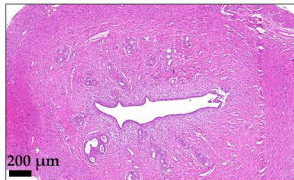

72 h

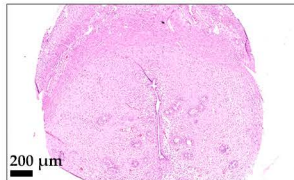

day 7

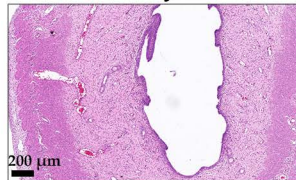

Supplement: Supplementary file 1 [file ijms-23-08808-s001.zip › ijms-1816572-figure S1.pdf]
